# Fatal Chloroform Narcosis Administered in the Operation of Teeth Extraction

**Published:** 1882-12-20

**Authors:** J. F. Byrn

**Affiliations:** Murfreesboro, Tenn.


### FATAL CHLOROFORM NARCOSIS ADMINISTERED IN THE OPERATION OF TEETH EXTRACTION.

By J. F. Byrn, M. D., Murfreesboro, Tenn.

On September 6th, Mrs. Temperance Smith and Mrs. Emma Smith, of this county, came into town, Mrs. Temperance Smith intending to have some teeth extracted. They called at my office about 12 o'clock, and asked me to accompany them to the dental office of Dr. Hartman; Mrs. Temperance Smith stated that she had some teeth she wished extracted by Dr. Hartman, and that she wished me to be present and administer to her chloroform. Mrs. Smith, aged thirty years, was the mother of five children, and was the wife of a highly respected citizen in this community. I at once attempted to dissuade her from taking the chloroform. I told her there was more danger from it when used in extracting teeth than any other operation; I said to her that I had almost ceased to use it for this purpose, and would consent to give it to her only upon the condition that she would assume all risk of danger. I assured her, after examining her teeth, that they could be extracted without much difficulty, and advised her to have it done without the use of chloroform. After these statements from me, she replied that she had taken it before several times for the purpose of having teeth extracted, and that Dr. Ransom, her family physician, had assured her that she could take it without any danger whatever. She said she was very well, and had been for sometime, and I observed that she was very cheerful and in good spirits. She insisted that she would take all risk of danger, and, indeed, apprehended none, so far as I could see. I agreed, therefore, to administer the chloroform, and in a few moments went to Hartman's office, they arriving there shortly before I did. After a few moments delay, Dr. Hartman arranged for Mrs. Smith to take the operating chair, another lady yielding it to her. I then stated to Dr. Hartman that she wished me to give her chloroform, and that I had advised her agai..st it very fully. He also joined with me in advising her not to take it, but she said to him that she had taken it in his office before on two different occasions for the same purpose; that she had not the least fear of a serious result from its use; that she was willing to assume the risk of any danger there was in taking it, and that she would not have her teeth extracted without using it. She then took the chair, preparing herself for the operation, by removing any undue pressure of her clothing. Dr. Hartman produced his bottle, containing about one ounce of chloroform. I administered the drug on a folded towel, and the total quantity given did not exceed two drachms. She took it well, and as the towel was withdrawn, asked for more chloroform, saying she did not have enough; but no more was given, and she passed rapidly under its influence, without passing through the usual stage of excitement. Her pulse and respiration were satisfactory, and Dr. Hartman at once extracted two teeth very quickly without trouble, and was preparing to take hold of the third one, when we noticed that her respiration was becoming embarrassed, and her eyes were wide open, fixed and staring; we removed her immediately from the chair, and laid her on the sofa and promptly applied the well-known restoratives. We dashed cold water upon the face and chest, depressed the head, raised the lower extremeties, pulled the tongue well forward, slapped the face with a wet towel, used brandy hypodermically, and kept up our efforts with artificial respiration for three-quarters of an hour; there was no galvanic battery to be had, therefore did not use it. Dr. R. S. Wendel being called, came at once, and assisted us. All our efforts to restore her were without avail. She never breathed after being laid on the sofa, and the heart ceased to beat in less than five minutes after she took the first inhilation of chloroform.

We append below the verdict of the coroner's jury in the above case:

State of Tennessee, Rutherford County.—An inquisition holden at Murfreesboro, in the county and State aforesaid, on the 6th day of September, 1882, before me, W. H. Blanch, Coroner of said county, upon the body of Mrs. Temperance Smith, there lying dead, by the jurors whose names are hereto subscribed, who upon their oaths do say, after hearing the testimony of the foregoing witnesses, to-wit: Mrs. Emma E. Smith, Mrs. Emma Penuel, Dr. J. F. Byrn, Dr. A. Hartman, Dr. J. B. Murfree, Dr. J. E. Wendel, Dr. H. H. Clayton, Francis Jane Gannaway, colored, that the said Mrs. Temperance Smith came to her death by the inhalation of chloroform, for the purpose of extracting teeth, administered by the hands of Drs. J. F. Byrn and Alex. Hartman; that the chloroform was administered at her own urgent request, and against the protestations of Drs. Byrn and Hartman.

We do furthermore find that the chloroform was administered with the utmost care and caution, and in appropriate quantities.

We furthermore find, from the examination of physicians, that chloroform is capable, and has produced death under similar circumstances, and that death is not due to any negligence in the administration of the chloroform, but it is the effect of the unknown peculiarities of the drug what is termed an accident. Mrs. Smith came to her death by the paralysis of the heart, produced by the inhalation of chloroform.

We furthermore find, that, in our opinion, Drs. Byrn and Hartman are free from any blame or censure in the death of Mrs. Smith, whatever.—*Nashville Jour. Med. and Surg.*

